# Low-Abundance Members of the *Firmicutes* Facilitate Bioremediation of Soil Impacted by Highly Acidic Mine Drainage From the Malanjkhand Copper Project, India

**DOI:** 10.3389/fmicb.2018.02882

**Published:** 2018-12-11

**Authors:** Abhishek Gupta, Avishek Dutta, Jayeeta Sarkar, Mruganka Kumar Panigrahi, Pinaki Sar

**Affiliations:** ^1^Environmental Microbiology and Genomics Laboratory, Department of Biotechnology, Indian Institute of Technology Kharagpur, Kharagpur, India; ^2^School of Bioscience, Indian Institute of Technology Kharagpur, Kharagpur, India; ^3^Department of Geology and Geophysics, Indian Institute of Technology Kharagpur, Kharagpur, India

**Keywords:** acid mine drainage, bioremediation, *Firmicutes*, biostimulation, quantitative PCR, metagenomics, dissimilatory sulfate reduction

## Abstract

Sulfate- and iron-reducing heterotrophic bacteria represented minor proportion of the indigenous microbial community of highly acidic, oligotrophic acid mine drainage (AMD), but they can be successfully stimulated for *in situ* bioremediation of an AMD impacted soil (AIS). These anaerobic microorganisms although played central role in sulfate- and metal-removal, they remained inactive in the AIS due to the paucity of organic carbon and extreme acidity of the local environment. The present study investigated the scope for increasing the abundance and activity of inhabitant sulfate- and iron-reducing bacterial populations of an AIS from Malanjkhand Copper Project. An AIS of pH 3.5, high soluble SO_4_^2−^ (7838 mg/l) and Fe (179 mg/l) content was amended with nutrients (cysteine and lactate). Thorough geochemical analysis, 16S rRNA gene amplicon sequencing and qPCR highlighted the intrinsic metabolic abilities of native bacteria in AMD bioremediation. Following 180 days incubation, the nutrient amended AIS showed marked increase in pH (to 6.6) and reduction in soluble -SO_4_^2−^ (95%), -Fe (50%) and other heavy metals. Concomitant to physicochemical changes a vivid shift in microbial community composition was observed. Members of the *Firmicutes* present as a minor group (1.5% of total community) in AIS emerged as the single most abundant taxon (∼56%) following nutrient amendments. Organisms affiliated to *Clostridiaceae, Peptococcaceae, Veillonellaceae, Christensenellaceae, Lachnospiraceae, Bacillaceae*, etc. known for their fermentative, iron and sulfate reducing abilities were prevailed in the amended samples. qPCR data corroborated with this change and further revealed an increase in abundance of dissimilatory sulfite reductase gene (*dsr*B) and specific bacterial taxa. Involvement of these enhanced populations in reductive processes was validated by further enrichments and growth in sulfate- and iron-reducing media. Amplicon sequencing of these enrichments confirmed growth of *Firmicutes* members and proved their sulfate- and iron-reduction abilities. This study provided a better insight on ecological perspective of *Firmicutes* members within the AMD impacted sites, particularly their involvement in sulfate- and iron-reduction processes, *in situ* pH management and bioremediation.

## Introduction

Acid mine drainage (AMD) is considered to be a global environmental problem faced by mining industries due to the biological oxidation of sulfidic minerals (Johnson and Hallberg, 2005; Neculita and Zagury, 2008; Qian et al., 2017). Owing to its highly toxic nature manifested through acidic pH, elevated levels of heavy metals and sulfate, AMD is not only a threat to aquatic and terrestrial ecosystems but considered to be a major contributor in long term degradation of environmental quality (Johnson and Hallberg, 2005; Chandra and Gerson, 2010; Hallberg, 2010). Despite its extreme nature, a diverse range of microorganisms inhabit AMD systems (Méndez-García et al., 2015; Chen et al., 2016; Huang et al., 2016). The most dominant bacterial populations residing in AMD are highly acidophilic, chemolithoautotrophic iron and sulfur oxidizers such as *Acidithiobacillus*, *Leptospirillum*, *Ferrithrix*, and *Ferritrophicum* etc. (Baker and Banfield, 2003; Chen et al., 2015, 2016; Méndez-García et al., 2015; Huang et al., 2016; Mesa et al., 2017; Teng et al., 2017). These acidophilic, autotrophic and Fe/S oxidizing microorganisms mainly contribute toward AMD generation and were studied extensively for their physiology, molecular mechanisms and ecological relevance (Denef et al., 2010; Kuang et al., 2013; Méndez-García et al., 2014; Chen et al., 2015; Goltsman et al., 2015; Chen et al., 2016), whereas the small heterotrophic populations thriving in the same niches could be of great significance in reducing AMD generation process and attenuating the overall hazard of these systems remain less explored.

Microbial sulfur- and iron-metabolisms through redox transformations coupled with or without energy generation constitute the major biochemical reactions within AMD (Baker and Banfield, 2003; Druschel et al., 2004). These transformation reactions facilitate generation of acidity and contribute toward raising the soluble -sulfate or -iron concentrations, while on the other hand could lead to reversal of such processes and aid to restoration of such environments. Sulfate- and iron-reductions are the two key reactions carried out by heterotrophic sulfate- or iron-reducing bacteria (SRBs or IRBs) that could reverse the AMD generation, metal precipitation and thus decrease the soluble metal concentrations and facilitate in raising the pH of AMD or AMD impacted ecosystems (Kaksonen et al., 2004; Church et al., 2007; Bijmans et al., 2009, 2010; Giloteaux et al., 2013). Bioremediation of AMD or AMD impacted ecosystems have been a subject of intense research in last decades (Kaksonen et al., 2004; Luptakova and Kusnierova, 2005; Church et al., 2007; Hiibel et al., 2008; Becerra et al., 2009; Bijmans et al., 2009; Hiibel et al., 2011; Burns et al., 2012; Moreau et al., 2013; Xingyu et al., 2013; Lefticariu et al., 2015; Sahinkaya et al., 2015; Deng et al., 2016; Zhang et al., 2016; Kefeni et al., 2017). In particular, enhancing the activities of indigenous microorganisms capable of sulfate- and/or iron-reduction and generation of alkalinity have gained interest for developing *in situ* bioremediation strategies (Neculita et al., 2007; Hiibel et al., 2008, 2011; Becerra et al., 2009; Bijmans et al., 2009; Burns et al., 2012; Xingyu et al., 2013; Lefticariu et al., 2015).

It is interesting to note that AMD or AMD impacted environment harbors SRBs and/or IRBs, but generally with low abundance and they remained metabolically less active at pH < 5.0 (Church et al., 2007; Sánchez-Andrea et al., 2011, 2012a; Giloteaux et al., 2013; Méndez-García et al., 2015). The limited presence and activities of these bacteria in AMD could be due to the presence of low organic carbon/other environmental variables and thermodynamic limitations as dissimilatory sulfate- and/or iron-reduction are energetically expensive (Church et al., 2007; Muyzer and Stams, 2008; Bird et al., 2011; Johnson, 2012; Giloteaux et al., 2013). Nevertheless, metabolic versatility of SRB has been exploited in bioremediation of AMD with different approaches, among which amendment of suitable carbon and electron sources, nitrogen, phosphorus compounds etc. are important (Kaksonen et al., 2004; Church et al., 2007; Neculita et al., 2007; Hiibel et al., 2008, 2011; Becerra et al., 2009; Bijmans et al., 2009; Burns et al., 2012; Xingyu et al., 2013; Zhang and Wang, 2014; Lefticariu et al., 2015; Zhang et al., 2017).

During the past decades, microbiology of AMD has been studied extensively, particularly the cultivation-independent deep sequencing studies have resolved the community composition and biogeochemical functions of previously unknown microorganisms (Bertin et al., 2011; Kuang et al., 2013; Méndez-García et al., 2014; Chen et al., 2015; Goltsman et al., 2015; Hua et al., 2015). In contrast, exploration of AMD communities with special reference to heterotrophic SRBs and IRBs or other metal reducing populations remained less explored (Giloteaux et al., 2013). *In situ* bioremediation of these hazardous wastes is limited due to paucity of knowledge on the diversity of SRBs/IRBs and factors that promote their activities.

In the present study we aimed to explore the abundance and role of indigenous sulfate- and/or metal-reducing bacterial populations in natural attenuation of an AMD impacted soil designated as AIS. Soil impacted with highly acidic, sulfate- and multiple heavy metal-rich AMD from Asia’s largest open-cast copper mine of Malanjkhand Copper Project (MCP) was used in this study. Microcosm based approach was adopted to promote presence and activities of indigenous sulfate- and/or metal-reducing bacteria using cysteine and lactate as biostimulation agents. A thorough assessment of microbial populations involved in sulfate/metal reduction and their characterization was done through 16S rRNA gene based amplicon sequencing coupled with qPCR and DGGE. The study was structured to answer the following questions: (i) How far it is possible to enhance the presence and activities of indigenous sulfate- and iron-reducing microbial populations present within an AMD impacted soil? (ii) What is the effect of such treatment(s) in the improvement of local physicochemical conditions, particularly the pH, concentrations of soluble -sulfate, -iron and -other heavy metals present therein? and (iii) Is it possible to enrich and cultivate the specific populations responsible for sulfate- and iron-reduction and management of the local physicochemical condition? The study demonstrates a comprehensive composition of microbial community residing in AIS and investigates the scope for *in situ* bioremediation.

## Materials and Methods

### Sampling Site

The AMD impacted soil was collected in a sterile container from 5–10 cm below the top layer of a field flooded with AMD from a neighboring sump of Malanjkhand Copper Project (MCP), Balaghat district, Madhya Pradesh, India (N 21° 59.91′, E 080° 41.879′) in the year 2014. The soil is exposed to AMD for over 10 years. The AMD water is released (as overflow) from the adjacent sump which receives AMD continuously from the mine areas. Selected physicochemical parameters such as oxidation reduction potential (ORP), pH and conductivity were measured on-site using multiparameter (Orion Star A329 portable Multiparameter, Thermo Fisher Scientific). All samples were collected following aseptic techniques, stored immediately at 4°C, brought to the laboratory and stored at -80°C till further processing.

### Microcosm Preparation

The microcosm setup was prepared with 5 g of AMD contaminated soil (AIS) using 20 ml filter sterilized distilled water in 30 ml glass vial. Three sets of microcosms were prepared. The first microcosm was amended with 0.1% (w/v) cysteine hydrochloride and designated as C. The second microcosm was amended with both 0.1% (w/v) cysteine hydrochloride and 0.1% (w/v) lactate (as sodium lactate), designated as C+L. The third microcosm was not amended with anything extra and designated as H (H stands for H_2_O, since only filter sterilized distilled water was present with AIS). Killed control was prepared for each setup by adding 2% (w/v) HgCl_2_ as biocide. The glass vials were sealed with gas-tight rubber stoppers and aluminum crimp seals. To mimic the natural environment nitrogen was not purged into the microcosm vials. The microcosms were incubated in dark for 180 days at 30°C. Each microcosm was set up in duplicate. Since the microcosms were of sacrificial type (i.e., the vial once opened was not reused in the same study) three experimental replicates were prepared: one for 4 months (120 days) incubation and marked as C_4M, C+L_4M, and H_4M; second for 5 months (150 days) incubation and marked as C_5M, C+L_5M, and H_5M and third for 6 months (180 days) incubation and marked as C_6M, C+L_6M, and H_6M. Physicochemical parameters were measured from each microcosm setups (at 120 and 180 days of incubation). Samples were withdrawn from each of the setup in triplicates and used for measuring the physicochemical parameters. The major physicochemical parameters such as pH and ORP of the slurry were measured by Orion Multi parameter (Orion Star A329 portable Multiparameter, Thermo Fisher Scientific). The slurry samples were taken out from the microcosm setup and centrifuged at 4000 rpm to settle down the soil particles. SO_4_^2−^ estimation was performed with the supernatant through BaCl_2_ turbidometric spectroscopy based method (Chesnin and Yien, 1951) while for Fe^2+^ estimation, samples were acidified to avoid any oxidation and Fe^2+^ concentration was measured by Ferrozine method (Viollier et al., 2000). The major elements such as Fe, Cu, As, Cr, Ni, and Zn were estimated from the slurry using atomic absorption spectroscopy (Perkin Elmer). In short, the slurry was centrifuged at 4000 rpm and supernatant was passed through 0.22 μm filter membrane and 2% HNO_3_ was added to prevent any oxidation.

### Metagenome Extraction, Library Preparation, and Sequencing

The microbial diversity analysis based on 16S rRNA gene amplicon targeted sequencing was performed with 6M setups (i.e., with 180 days incubation). Original AIS sample (0_Day) was also used for comparison. From the three microcosms and the 0_Day AIS, samples were withdrawn in triplicates and metagenome was extracted from each of the withdrawn samples using Power Soil DNA Isolation Kit (MoBio laboratories) according to the manufacturer’s protocol. Metagenome from the replicate samples were pooled, mixed thoroughly and used for amplification of V4 region of 16S rRNA gene. V4 region of 16S rRNA gene was amplified with V4 specific primers (Bates et al., 2011). The following amplification conditions: 95°C for 5 min, 35 cycles of 95°C for 40 s, 50°C for 45 s and 72°C for 40 s with final extension at 72°C for 7 min were used for amplification of V4 region. Thereafter amplicons were purified using 2% E-gel (E-Gel SizeSelect II Agarose Gel, Thermo Fisher Scientific) and sequencing was performed with Ion S5^TM^ System (Thermo Fischer Scientific). In order to understand the microbial diversity at 5M setups (i.e., with 150 days incubation), Denaturing gradient gel electrophoresis (DGGE) was performed with H_5M, C+L_5M, and C_5M samples. Metagenome was extracted in triplicates from these setups and were pooled together to amplify the V4 region using GC-clamp forward primer as described above. A DCode Universal Mutation Detection system (Bio-Rad, United States) was used to perform DGGE with similar protocol as described by Paul et al. (2015). The denaturing gradient from 35 to 70% was used for the present study. Twenty-three distinct bands in DGGE profile were excised and eluted by keeping it in 20 μl DNase free PCR water at 4°C for overnight. These gel eluted products were re-amplified by using without GC clamp 515F and 806R primers (V4 region) and were cloned into the pTZ57RT vector for sequencing. EzTaxon ^1^ and SILVA 119 reference database ^2^ were used for the taxonomic assignment of the obtained sequences.

### Quantification of Bacterial/Specific Taxa and *dsr*B Copy Number

Quantification of bacterial abundance and remarkably shifted taxa; *Firmicutes*, *Acidobacteria*, *Actinobacteria* as well as *dsr*B gene involved in sulfate reduction were performed for all the samples (0_Day, H_6M, C_6M, and C+L_6M). The bacterial abundance was quantified through bacterial specific 16S rRNA gene copy number. Similarly, abundance of *Actinobacteria*, *Acidobacteria*, and *Firmicutes* were quantified through specific 16S rRNA gene specific to these taxa. Copy numbers of functional gene *dsr*B were also quantified using qPCR based technique to estimate the sulfate-reducing populations. Real-time primers for bacterial 16S rRNA gene was taken from Muyzer et al. (1993), primers specific to *Actinobacteria* and *Firmicutes* was taken from Mühling et al. (2008), primer used for *Acidobacteria* as described by Lee and Cho (2011) and *dsr*B was taken from Purkamo et al. (2013). The qPCR was performed in Quant Studio 5 Real-Time PCR System (Thermo Fisher Scientific) with Power SYBR green PCR Mastermix (Invitrogen), with a total volume of 10 μl containing primer concentration of 5 picomoles and 2 μl of metagenomic DNA. All the reactions were set in triplicates. The following amplification conditions: 95°C for 10 min, 40 cycles of 95°C for 15 s, 55°C for 30 s and 72°C for 30 s was followed for bacterial and *d*srB gene while 63°C, 59°C, and 57°C annealing temperature were used for *Actinobacteria, Acidobacteria*, and *Firmicutes*, respectively. Melting curve analysis was run after each assay to check PCR specificity. Bacterial 16S rRNA gene copy numbers were determined in each sample by comparing the amplification result to a standard dilution series ranging from 10^2^ to 10^8^ of plasmid DNA containing the 16S rRNA gene of *Achromobacter* sp. MTCC 12117. *Firmicutes* gene copy number was calculated from plasmid DNA containing 16S rRNA gene from *Bacillus*. Whereas 16S rRNA gene of *Actinobacteria* and *Acidobacteria* as well as *dsr*B gene were cloned from metagenome and different dilution series of plasmid DNA copy number were used to prepare the standard curve for comparing the amplification result. The efficiency of qPCR was calculated using formula E = 10 (−1/ − Slope) – 1. The standard curve was linear for all the taxa specific and *dsr*B gene. *R*^2^ value was greater than 0.993 for all the standard curve while efficiency was ranges from 84 to 112% (Supplementary Table S1).

### Enrichment of *Firmicutes* Specific Members and Their Potential Role in Fe^3+^ and SO_4_^2−^ Reduction

*Firmicutes* specific populations were enriched in facultative anaerobic medium (Stieglmeier et al., 2009) and *Clostridium* specific medium containing following ingredients in g/L NaCl 2.0, K_2_HPO_4_ 5.0, MgCl_2_ 0.2, ferric citrate 0.2, yeast extract 1.0, lysine 0.5 and cellulose 7.0 at pH 7.0 in 50 ml glass serum vials. Both the media were purged with filtered N_2_ gas for 15–20 min to remove the oxygen and cysteine HCl (0.025%) was added as a mild reducing agent. Serum bottles were sealed with rubber stoppers. Two ml slurry from both C_6M and C+L_6M was used as inoculum in both the media and incubated at 30°C for 2 weeks. The enrichment was sub-cultured three times in the same media before transferring into sulfate reducing medium (SRM) (modified from Postgate, 1963) and iron reducing medium (IRM) (containing ferric citrate 5 mM, NH_4_Cl 1.50 g/L, NaH_2_PO_4_ 0.60 g/L, KCl 0.10 g/L, sodium acetate 2.50 g/L and yeast extract 0.05 g/L). Nitrogen gas was flushed for 15–20 min and cysteine HCl (0.025%) was added as a mild reducing agent in both the media to make the environment anaerobic. The pH of these two media was set to 7.0 using 1N NaOH/1N HCl and incubated at 30°C for 2 weeks. Enriched population was sub-cultured thrice in same media after seeing the visual changes in the media (iron containing medium turned colorless, sulfate reducing medium turned black due to precipitation of iron sulfide). Remaining sulfate and increased iron (Fe^2+^) concentrations were measured for assessing the reduction of sulfate and iron (Fe^3+^) using BaCl_2_ turbidometric method and Ferrozine method, respectively. Briefly, 2 ml samples were taken out and bacterial cells were pelleted down to use supernatant for estimation of SO_4_^2−^ and Fe^2+^ concentration.

### DNA Extraction From Enrichment

Total DNA from enriched populations was extracted from 4 ml of each enrichment. Equal volume of 0.5 M ammonium oxalate was added in iron enrichment to dissolve iron precipitates. The culture was pelleted at high speed for 5 min at room temperature. The cell pellet was dissolved in 500 μL TNE buffer (Tris HCl-10 mM, NaCl-2.0 M, EDTA-1 mM), 1/10 volume silica bead was added and vortexed for 15–20 min. 100 μL lysozyme (100 mg/ml) was added in the cell suspension, vortexed briefly to mix and incubated at 37°C for 2 h. 30 μL proteinase K (20 mg/ml) and 50 μL SDS (10%) were added and incubated at 37°C for 45 min. DNA was then extracted using chloroform:isoamyl alcohol (24:1). DNA pellet was washed twice with ice-cold 70% ethanol and the pellet was air dried. DNA was resuspended in PCR grade water. 16S rRNA gene amplicon from the DNA was prepared as described above for microcosm treatments (see section “Metagenome Extraction, Library Preparation, and Sequencing”). To understand the microbial diversity of these enrichments, amplicon based analysis was performed with *Clostridium* and facultative enrichments from both C_6M and C+L_6M setups but to identify the main iron and sulfate reducing populations, enrichments from C_6M was considered.

### Diversity Analysis and Statistical Tool

Ion Torrent data analysis of V4 region of 16S rRNA gene was performed with QIIME 1.9.1 pipeline (Caporaso et al., 2010). Quality filtering of reads and bioinformatics were performed as described by Gupta et al., 2017. In brief, quality filtering was performed for raw reads to remove primers, sequences with homopolymers run of >6 bp and read length beyond the range of 230–300 bp. Only 3 primer mismatches were allowed due to degeneracy of primer set in this step. Denovo OTU picking was performed with uclust and SILVA 119 reference database ^3^ was used for taxonomy assignments of reads as mentioned in QIIME pipeline. The OTU level analysis was performed by sub-sampling the samples to the lowest number of reads obtained in any of the samples through QIIME 1.9.1 pipeline. Venn diagram was generated in InteractiVenn^4^ (Heberle et al., 2015) for top 100 OTUs. Microbial metabolic pathways were estimated based on the 16S rRNA gene data from the closed OTU picking method using PICRUSt software package (Langille et al., 2013) on the web-based Galaxy server^5^. For PICRUSt analysis, Greengenes database^6^ was used for taxonomy assignment. One-way ANOVA was performed to assess the changes in the microbial diversity between the treatments using PAST software version 3.20 (Hammer et al., 2001). Weighted pair group mean arithmetic (WPGA) based hierarchical clustering was performed with Bray–Curtis distance dissimilarity matrix. Ternary plot was generated using PAST software to assess difference in diversity pattern among the treatments. All the data represented for physicochemical parameters were mean of its triplicates with standard deviation.

### Nucleotide Accession Number

Metagenomic sequences are available under the NCBI BioProject ID PRJNA416924. The SRR number for each samples are SRR6320797 (C+L_6M), SRR6320796 (C_6M), SRR6320800 (0_Day), SRR6320884 (H_6M), SRR6320885 (FA_C_6M), SRR6320921 (Clos_C_6M), SRR6320922 (Clos_IRM), SRR6320919 (FA_IRM), SRR6320923 (Clos_SRM), SRR6320920 (FA_SRM), SRR7865998 (FA_C+L) and SRR7865999 (Clos_C+L). Sequence of DGGE bands were submitted in Genbank under accession numbers MH938427-MH938447.

## Results

### Change in Physicochemical Parameters After the End of Incubation

Nutrient amendments to AIS facilitated a considerable improvement of its physicochemical conditions (Table 1). At the onset of the study (0_Day), major physicochemical parameters of the soil slurry were measured. This sample was found to be of highly acidic (pH 3.51) nature; rich in soluble SO_4_^2−^ (7838 mg/l) and Fe (179 mg/l). Following incubation with nutrients, significant increase in pH (up to pH 6.61) but decrease in ORP (up to 110 mV) were observed coupled with considerable changes in concentrations of SO_4_^2−^, Fe, Fe^2+^ and heavy metals. Control set (H_6M) with only water addition showed slight change with respect to the test physicochemical parameters while killed control did not show any shift at all. Incubation with only water (H_6M) could initiate reactions responsible for the observed shift in pH and ORP, presence of nutrients favored such reactions significantly. Following cysteine and cysteine + lactate amendment, soluble sulfate concentration was greatly reduced along with Fe (total Fe as well as Fe^2+^), Cu, Zn and Ni. Compared to H_6M that showed nearly 50% decrease in SO_4_^2−^ (to that of its initial level), cysteine + lactate addition could led to a 95% reduction. Microcosm amended with only cysteine showed only up to 76% lowering of SO_4_^2−^ (compared to 0_Day). Soluble Fe level presented an interesting trend: concentrations of both total Fe and Fe^2+^ were enhanced in H_6M (2.3-fold for Fe and 2.5-fold Fe^2+^) and C_6M (5.6-fold for Fe and 4.8-fold for Fe^2+^), while the values decreased significantly (0.5-fold for Fe and 0.6-fold for Fe^2+^) in C+L_6M. Although an overall enrichment experiment showed a strong role of the test nutrients in improving the local physicochemical condition of the AMD impacted soil, lactate + cysteine was identified as a better stimulant than cysteine alone.

**Table 1:** Details of physicochemical parameters of the microcosm setup.

Parameters	0_Day	H_4M	H_6M	C_4M	C_6M	C+L_4M	C+L_6M
pH	3.51 ± 0.01	3.60 ± 0.01	4.01 ± 0.01	5.86 ± 0.02	6.37 ± 0.01	6.12 ± 0.01	6.61 ± 0.01
ORP	200.51 ± 0.95	165.7 ± 1.0	140.61 ± 1.55	130.02 ± 1.09	120.23 ± 1.20	125.21 ± 1.15	110.02 ± 1.01
SO_4_^2−^	7838.20 ± 39.64	6780.78 ± 54.08	4005.88 ± 19.15	4282.33 ± 22.72	1860.21 ± 14.75	720.06 ± 11.31	365.58 ± 22.11
Fe^2+^	130.89 ± 4.72	202.81 ± 3.57	336.64 ± 12.66	980.37 ± 6.45	628.83 ± 9.08	300.64 ± 4.77	79.29 ± 2.1
Fe	179.10 ± 1.55	300.81 ± 1.0	415.75 ± 1.75	1386.31 ± 1.11	735.13 ± 0.89	320.94 ± 1.10	90.01 ± 1.10
Cu	1.84 ± 0. 07	0.31 ± 0.11	0.28 ± 0.08	0.13 ± 0.06	0.12 ± 0.07	0.18 ± 0.06	0.16 ± 0.05
Zn	1.79 ± 0.33	0.57 ± 0.21	0.46 ± 0.10	0.39 ± 0.10	0.16 ± 0.06	0.14 ± 0.04	0.13 ± 0.05
Ni	0.39 ± 0.11	0.32 ± 0.03	0.28 ± 0.06	0.16 ± 0.06	0.14 ± 0.07	0.16 ± 0.06	0.16 ± 0.07

*All the units are represented in ppm except ORP (mV) and pH (SI unit). H, C and C+L denote unamended, cysteine amended and cysteine and lactate amended microcosms while 4M (120 days incubation) and 6M (180 days incubation) represent the time of incubation. The values are represented mean of three independent experiments with standard deviations.*

**Table 2:** Details of 16S rRNA gene reads and non-parametric diversity indices of microbial communities from microcosms and enrichments.

Sample name	Raw reads obtained	Quality filtered reads	Chao1	Observed OTUs	Simpson’s index	Shannon’s index	Goods Coverage
0_Day^∗^	853984	516037	33511	17237	0.93	6.38	0.98
H_6M^∗^	1613332	945208	59309	30274	0.97	7.45	0.98
C_6M^∗^	1951074	1444246	51674	28392	0.93	6.36	0.99
C+L_6M^∗^	1011838	590466	46528	23116	0.93	6.95	0.98
FA_C_6M	1299817	904305	16863	9003	0.51	2.72	0.99
FA_SRM^∗∗^	1616369	1321436	33076	16041	0.41	2.71	0.99
FA_IRM^∗∗^	1913139	1597931	32597	16545	0.36	2.44	0.99
Clos_C_6M	143284	95425	11341	5439	0.90	6.00	0.97
Clos_SRM^∗∗^	1075968	846637	15109	8178	0.41	2.50	1.00
Clos__IRM^∗∗^	299768	231499	14908	7311	0.60	4.23	0.98
FA_C+L_6M	703433	349793	4135	2725	0.48	2.71	0.99
Clos_C+L_6M	498707	381545	6810	4015	0.54	2.86	0.99

*^∗^Denotes the microcosm setup while rest denotes the enrichments. Enrichment was performed from both the setup (C_6M and C+L_6M) in facultative anaerobic and Clostridium specific media. FA, facultative anaerobic enrichment; FA_SRM, facultative anaerobic enrichment in sulfate reducing medium; FA_IRM, facultative anaerobic enrichment in iron reducing medium; Clos, Clostridium specific enrichment; Clos_SRM, Clostridium specific enrichment in sulfate reducing medium; and Clos_IRM, Clostridium specific enrichment in iron reducing medium. ^∗∗^Denotes the enrichment was performed for C_6M.*

### Shift in Microbial Community Composition

**FIGURE 1:**
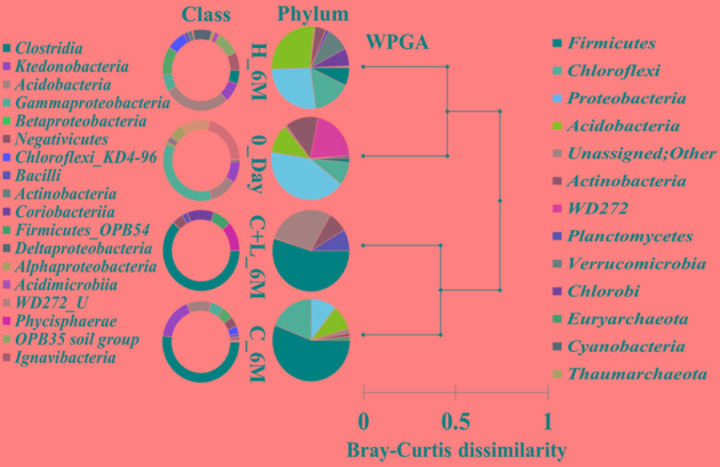
Distribution of relative abundance of 16S rRNA gene sequences detected in microcosm setup (amended and unamended) at phylum-level (cumulative abundance > 0.1%) and class-level (top 18) taxonomic resolution. WPGA based agglomerative hierarchical clustering of treatments on the basis of abundance of phylum using Bray–Curtis dissimilatory matrix is represented. U denotes uncultured member.

16S rRNA gene amplicon sequencing and estimated diversity indices revealed an assessable shift in microbial community composition of AIS following incubation with nutrient amendments (Table 2). Both estimated Chao1 and observed OTUs were increased coupled with distinct shifts in microbial community composition (Table 2). The most abundant bacterial phyla within the AIS at 0_Day were *Proteobacteria* (42%), WD272 (20%), *Actinobacteria* (14%), *Acidobacteria* (11%), *Chloroflexi* (9%), and *Firmicutes* (1.5%) (Figure 1). Following incubation, a distinct shift in community composition with great enhancement of *Firmicutes* coupled with the striking decrease in abundance of *Proteobacteria*, *Acidobacteria*, and *Actinobacteria* were detected (Figure 1). Abundance of the members of *Firmicutes* affiliated to *Clostridia*, OPB54, *Negativicutes*, and *Bacilli* was increased in both C+L_6M and C_6M. The extent of enhancement of *Firmicutes* was up to 36.5-fold in C_6M and 35.4-fold in C+L_6M (Figure 1). *Proteobacteria* [*Gammaproteobacteria* (35%)*, Alphaproteobacteria* (6%*)*, *Betaproteobacteria* (1%), and *Deltaproteobacteria* (0.07%)] that constituted the major phylum at 0_Day was found to be considerably less prevalent within the communities enriched with various amendments (Figure 1). The noteworthy decrease in abundance of *Gammaproteobacteria* and *Alphaproteobacteria* was observed in all the setup whereas abundance of *Betaproteobacteria* and *Deltaproteobacteria* was increased in C_6M (Figure 1). Members of the phylum *Chloroflexi* (*Ktedonobacteria* and KD4-96) also showed a substantiate increase in their abundance in C_6M (19.0%) while it got reduced in C+L_6M (0.03%). The other major classes such as *Acidobacteria*, *Acidimicrobiia, Actinobacteria*, and *Thermoleophilia* showed decrease in their abundance in C_6M and C+L_6M (Figure 1).

**FIGURE 2:**
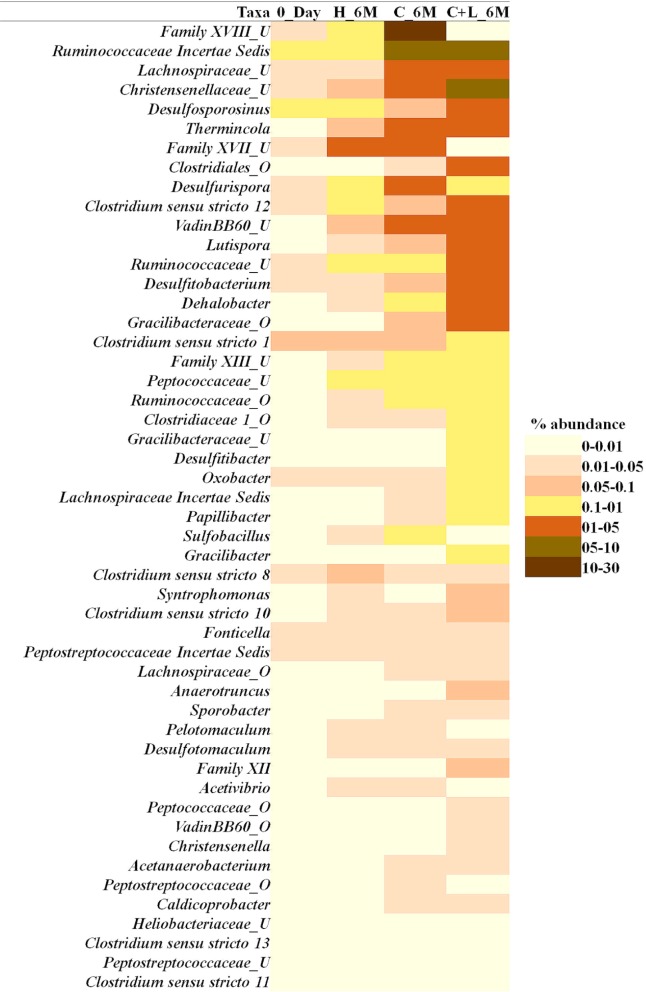
Heat map based relative abundance of distribution of top 50 *Clostridiales* members in all the microcosm setup. U and O denote uncultured member and others, respectively.

**FIGURE 3:**
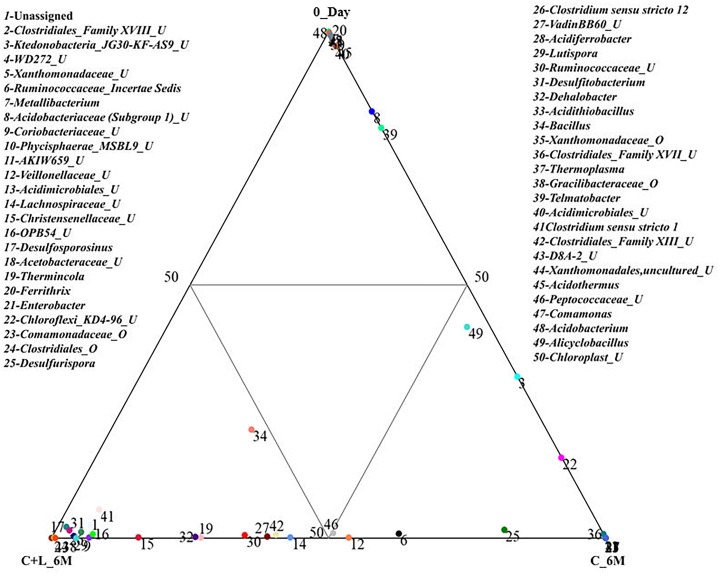
Distribution of taxonomic groups at genera level in 0_Day, C_6M and C+L_6M. The 50 most abundant genera (cumulative abundance > 0.2%) associated with each sample is visualized in ternary plots. The position in the triangle indicates the relative abundance of each taxon among the three samples. U and O denote uncultured member and others, respectively.

Family level analysis within the 0_Day, H_6M, C_6M and C+L_6M microcosms showed increase in abundance of several families. The abundance of facultative and/or strict anaerobic members of *Clostridiales* (*Clostridiaceae 1*, Family XIII, *Christensenellaceae*, *Lachnospiraceae*, *Gracilibacteraceae*, *Peptococcaceae*, *Peptostreptococcaceae*, *Ruminococcaceae*, and VadinBB60), *Bacillales* (*Alicyclobacillaceae* and *Bacillaceae)*, *Veillonellaceae*, uncultured OPB54 and *Coriobacteriaceae* was increased in both C_6M and C+L_6M. Heat map analysis (Figure 2) of the distribution of major genera (also considering taxa classified up to family level) under *Clostridiales* indicated considerable enhancement in abundance of several taxa commonly attributed to sulfate- and iron-reduction following nutrient amendments. In contrast to this, only water amendment (control; H_6M) allowed enhancement of mostly known taxa involved in iron and sulfur oxidation [*Acidobacteriaceae* (Subgroup 1), *Gallionellaceae*, *Xanthomonadaceae*] and few other taxa such as OPB35 soil group, KD4-96, *Ktedonobacteria*_JG30-KF-AS9, BSV26 and *Cystobacteraceae.* Overall the successful enrichment of diverse fermentative and anaerobic populations was achieved, suppressing the growth of acidophilic members following creation of anoxic environment and supply of metabolizable C- and N-sources. A ternary plot was generated to understand the distribution of top 50 genera across 0_Day, C_6M and C+L_6M microcosm samples (Figure 3). The result showed that acidophilic genera such as *Ferrithrix, Metallibacterium, Acidobacterium*, uncultured –*Acidomicrobiales* and *-Acidobacteriaceae* Subgroup 1 etc. were more prevalent at 0_Day. In contrast, taxa affiliated to *Firmicutes*; *Desulfitobacterium, Clostridium Sensu Stricto 1, Desulfosporosinus, Desulfurispora*, uncultured -*Christensenellaceae*, -OPB54, *-Clostridiales* Family XVII, *-Ruminococcaceae*, -Lachnospiraceae, *-Peptococcaceae* etc. capable of sulfate- and iron-reduction dominated in C_6M and C+L_6M. One-way ANOVA analysis confirmed that microbial diversity among the treatments was significantly different (*P* < 0.05).

### Microbial Shift at OTUs Level

**FIGURE 4:**
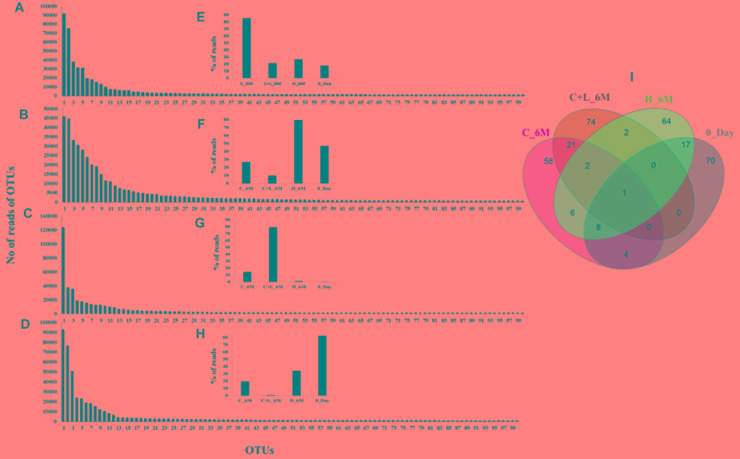
Rank abundance based analysis of top 100 OTUs in the microcosm setup. Rank abundance profile of top 100 OTUs from each sample is depicted **(A)** C_6M, **(B)** H_6M, **(C)** C+L_6M, and **(D)** 0_Day. **(E–H)** represent the percent distribution of top 100 OTUs across the samples. (I) Venn diagram shows the shared and unique OTUs across the samples (considering top 100 OTUs from each sample). Detail taxonomic affiliations of 100 OTUs are presented in Supplementary Table S2

**FIGURE 5:**
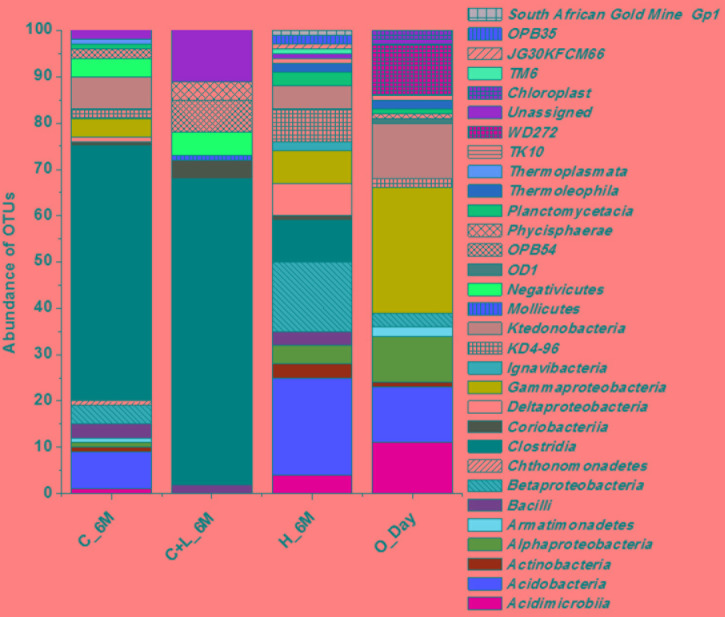
Taxonomic distribution pattern (at class-level) of top 100 OTUs in each setup.

**FIGURE 6:**
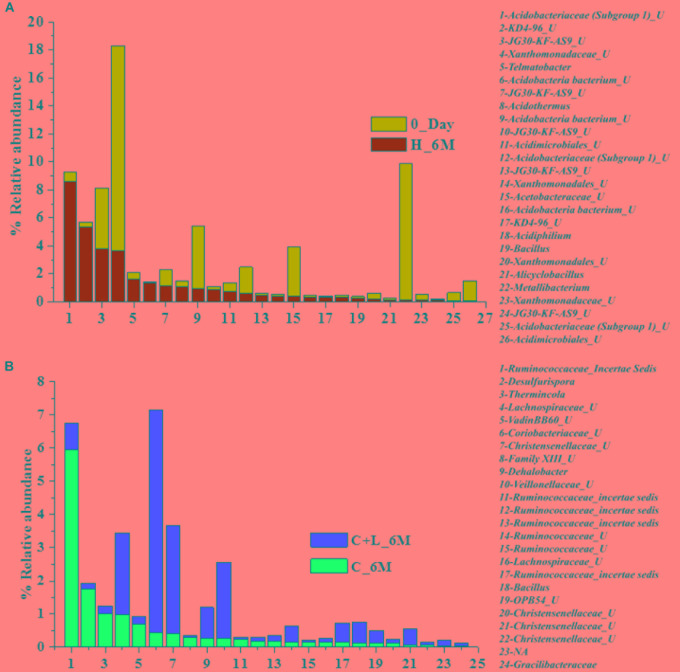
Distribution of shared OTUs (from top 100 OTUs) between the samples **(A)** 0_Day and H_6M and **(B)** C+L_6M and C_6M. U denotes uncultured member.

In order to understand the dynamics of microbial community composition beyond the taxonomic level, most abundant OTUs (top 100 OTUs) from each of the microcosms were analyzed (Figures 4A–D). Top 100 OTUs from each microcosm contributed 79–85% of the total reads of the respective samples. The interesting finding was that when considering top 100 OTUs of one treatment, the same OTUs in another treatment contributed less percentage of the total reads, clearly indicating the effect of treatments (Figures 4E–H). Venn diagram depicted the pattern of sharing of OTUs among the treatments (Figure 4I) and signified that how the abundance of OTUs was significantly changed during the treatments. Taxonomic identities of these OTUs were determined to find their affiliation to 32 different taxa (Figure 5). Out of 100 OTUs from each of the microcosms, OTUs affiliated to *Firmicutes* were dominant in C+L_6M (80 OTUs) and C_6M (64 OTUs) while OTUs assigned to *Proteobacteria* were high in 0_Day (40 OTUs) and H_6M (33 OTUs) (Figure 5). These results were perfectly in line with our taxonomy based observation of increasing abundance of *Clostridia* in C+L_6M and C_6M. Total 26 OTUs (out of top 100 OTUs) were found to be shared between 0_Day and H_6M (Figure 6A). These common OTUs were affiliated mostly to acidophilic taxa. Among the C_6M and C+L_6M communities 24 shared OTUs were detected and these were affiliated to iron/sulfate reducing, fermentative and anaerobic *Firmicutes* taxa (Figure 6B).

### qPCR Based Quantification of Bacterial/Specific Taxa and *dsr*B Gene

**FIGURE 7:**
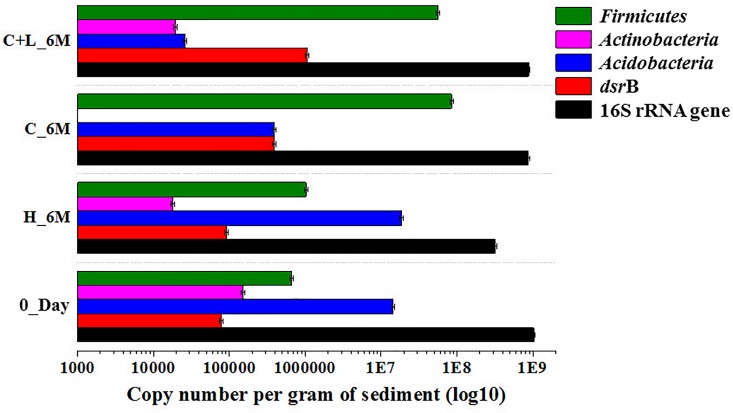
Quantitative PCR based analysis of gene copy number of bacteria/specific taxa and *dsr*B gene among the microcosm setup.

Quantitative estimation of the major taxa (*Firmicutes*, *Acidobacteria*, and *Actinobacteria*) as well as *dsr*B gene (involved in dissimilatory sulfate reduction) was performed for 0_Day, C_6M, C+L_6M and H_6M communities using qPCR based approach. Total bacterial 16S rRNA gene copies indicated a marginal reduction in bacterial abundance following microcosm amendments (Figure 7). The estimation of 16S rRNA gene copies for *Actinobacteria*, *Acidobacteria*, and *Firmicutes* corroborated with the amplicon based community data suggesting the decrease in abundance of *Actinobacteria* and *Acidobacteria* but increase in *Firmicutes* following nutrient amendment (Figure 7). The involvement of sulfate reducing bacteria in nutrient amended microcosms was highlighted by a remarkable increase in *dsr*B gene copy number from 7.8 × 10^4^ to 3.9 × 10^5^–1.0 × 10^6^ (Figure 7).

### PICRUSt Based Functional Prediction of the Community

Metabolic functions of the microbial communities were established through PICRUSt analysis. Using the genome-wide analysis tools integrated in PICRUSt, we could look into the genomic inventories related to sulfate and cysteine metabolism and other major biogeochemical processes of the enriched communities (Supplementary Figure S1). The result showed abundance of genes involved in dissimilatory sulfate metabolism (*apr*AB and *dsr*AB), cysteine metabolism (cysteine desulfhydrase, cysteine synthase, cystathionine synthase and cystathionine lyase) along with hydrogenases, metal tolerance/transporter gene for As, Fe, Cu, Zn, Co, etc., nitrogen metabolism and other major categories of metabolic functions. Considerable change in the abundance of *dsr*AB (involved in dissimilatory sulfate reduction) and cysteine desulfhydrase (involved in cysteine utilization) was observed in nutrient amended microcosms. The analysis clearly indicated that enriched microbial populations were genetically equipped for dissimilatory sulfate reduction following cysteine amendment.

**FIGURE 8:**
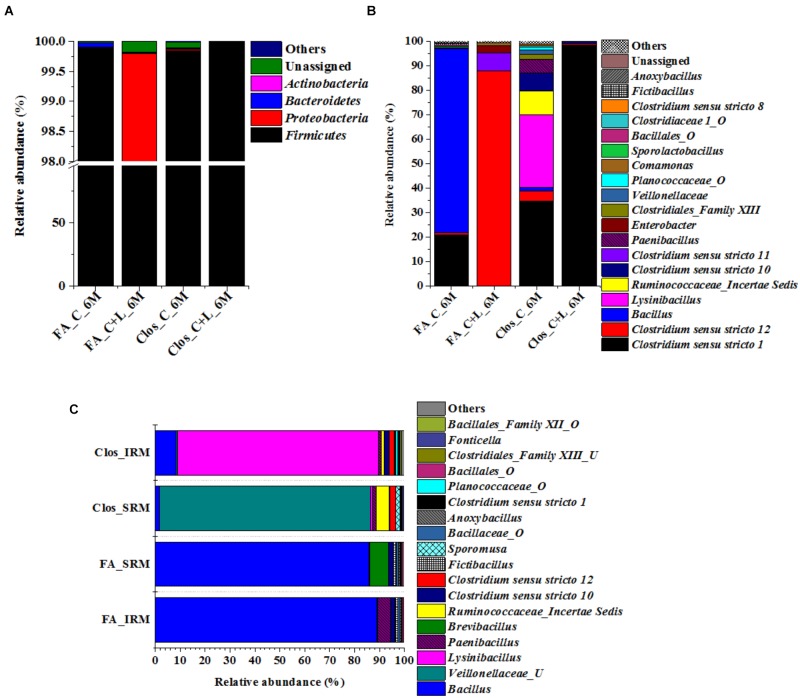
Taxonomic distribution of enrichment setups from C_6M and C+L_6M. **(A)** Phylum-level distribution of enriched populations in *Clostridium* specific (Clos) and Facultative anaerobic (FA) enrichment. **(B)** Distribution of genera in *Clostridium* specific enrichment and Facultative anaerobic enrichment. **(C)** Shift in microbial diversity (represented at genera-level) when enriched population of Clos_C_6M and FA_C_6M were subjected to iron and sulfate reducing medium. Clos_IRM: distribution pattern of *Clostridium* specific enrichment in iron reducing medium; Clos_SRM: distribution pattern of *Clostridium* specific enrichment in sulfate reducing medium; FA_IRM: distribution pattern of Facultative anaerobic enrichment in iron reducing medium and FA_SRM: distribution pattern of Facultative anaerobic enrichment in iron reducing medium. U and O denote uncultured member and others, respectively.

### Enrichment of *Firmicutes* Members Using Specific Medium From Microcosm Setup

The 16S rRNA gene based investigation indicated that following cysteine amendment abundance of members of *Firmicutes* were enriched considerably. Most of these taxa were known for their role in anaerobic sulfate- and iron-reduction. Although the qPCR and PICRUSt supported the role of these members in the observed physicochemical changes within our AIS microcosms, final validation of their biogeochemical role was done by enrichment of the *Firmicutes* members under specific culture conditions. Cultures from both C_6M and C+L_6M microcosms were sub-cultured in two specific media: *Clostridium* specific and facultative anaerobic. Following three repeated sub-culturing in the respective media, taxonomic identities of the enriched populations were established by 16S rRNA gene based amplicon sequencing (Figure 8A). The results indicated that our culture conditions were highly supportive for the enrichment of *Firmicutes* members in both the *Clostridium* specific and facultative anaerobic media. Members of this phylum contributed 95.33–99.85% while *Proteobacteria*, *Actinobacteria*, and *Bacteroidetes* constituted very small populations (Figure 8A). The most dominant genera detected in the *Clostridium* specific enrichment belonged to *Clostridiales* and *Bacillales* members such as *Clostridium sensu stricto 1, Lysinibacillus, Ruminococcaceae_incertae sedis, Clostridium sensu stricto 10, Clostridium sensu stricto 12, Paenibacillus, Clostridiales*_Family XIII, uncultured *Planococcaceae* and uncultured *Veillonellaceae* members (Figure 8B). In the facultative anaerobic medium sub cultured from C_6M microcosm, *Bacillus* was the most dominant genera (75.70%) followed by *Clostridium sensu stricto 1* (20.63%), *Clostridium sensu stricto 12* (1.01%) and *Sporolactobacillus* (0.68%) (Figure 8B). In contrast, *Clostridium sensu stricto 12* and *Clostridium sensu stricto 11* accounted for more than 95% of the community grown in the same medium but subcultured from C+L_6M (Figure 8B).

### Sulfate- and Iron-Reduction Potential of the Microorganisms Enriched in *Clostridium* Specific and Facultative Anaerobic Media

**FIGURE 9:**
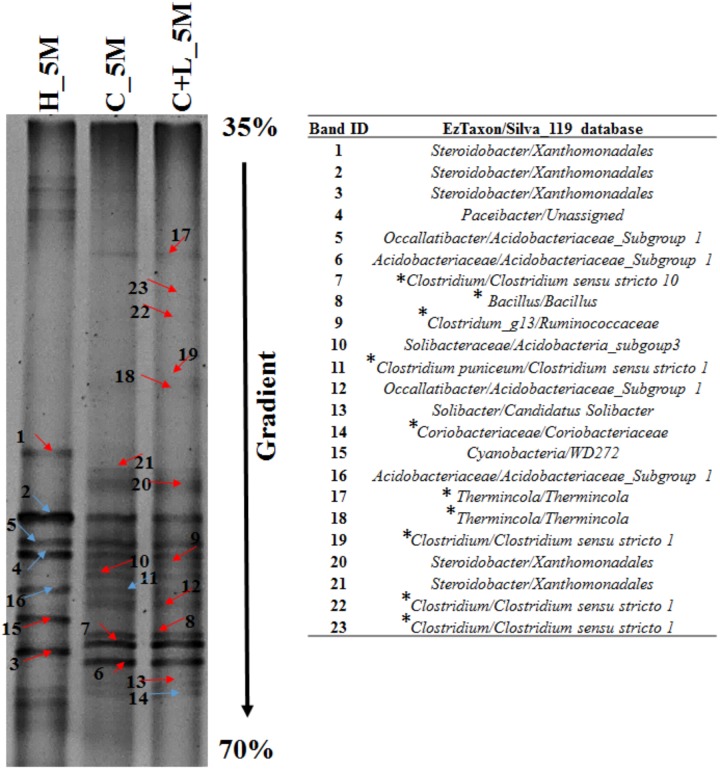
Denaturing gradient gel electrophoresis (DGGE) profile of treatments (H_5M, C_5M, and C+L_5M) at 150 days of incubation. Red arrow indicates distinct band while blue represents common bands. ^∗^Represents the organisms detected in both treatment and enrichment of C_6M and C+L_6M.

Bacterial cultures enriched in *Clostridium* specific and facultative anaerobic media were further inoculated in sulfate reducing and iron reducing media (designated as SRM and IRM) for assessing their potential toward sulfate- and iron-reduction. Following three repeated sub-culturing, 16S rRNA gene amplicon sequencing was done for all the four sets derived originally from C_6M microcosm. The amplicon sequencing result of *Clostridium* specific enrichment grown in SRM showed that uncultured *Veillonellaceae* members, *Ruminococcaceae incertae sedis* and *Clostridium sensu stricto 12* accounted for 92.25% (Figure 8C). The same enrichment culture grown in IRM showed the abundance of *Lysinibacillus* (80.43%) along with *Bacillus*, uncultured *Planococcaceae* member, *Clostridium sensu stricto 12* and *Clostridium sensu stricto 10* (Figure 8C). Similar study performed with facultative anaerobic enrichment indicated proliferation (cumulative abundance of 98%) of *Bacillus*, *Brevibacillus*, C*lostridium sensu stricto 10*, *Fictibacillus, Anoxybacillus*, and *Clostridium sensu stricto 1* in SRM (Figure 8C) while *Bacillus, Paenibacillus*, *Clostridium sensu stricto 10* and *Fictibacillus* accounted for 97.71% of the IRM culture (Figure 8C). Metabolic abilities of the enriched populations derived from microcosms C_6M and C+L_6M microcosms toward sulfate- and iron-reduction were confirmed by quantitative estimation of SO_4_^2−^ and Fe^2+^ ions. Nearly complete reduction of SO_4_^2−^ (15 mM) and Fe^3+^ (5 mM) were noticed following 10–14 days of incubation, confirming their abilities for reduction of these terminal electron acceptors. The formation of black precipitates of iron sulfide in SRM and change in color of the IRM from yellow to light green or colorless with precipitation of Fe (Ferric citrate as redox indicator) (Pan et al., 2017) was also noted (Supplementary Figure S2). In order to confirm the presence of these SRB and IRB after 4 months of incubation where a decline in soluble iron concentration (Fe^2+^) was observed due to precipitation with sulfide produced by sulfate reducing activity in the treatments, DGGE based microbial community analysis was performed with 5 months incubated microcosms (H_5M, C_5M, and C+L_5M). The banding pattern obtained for C_5M and C+L_5M communities showed enrichment of almost similar types of microbial populations (Figure 9). The enrichment of *Clostridium* sp., *Themincola* sp., *Bacillus* sp., *Steroidobacter* sp., as well as members of *Acidobacteriaceae*, *Ruminococcaceae*, and *Coriobacteriaceae* in these treatments clearly indicated their potential toward both iron- and sulfate-reduction (Figure 9). These groups were also detected in the same treatments through amplicon based sequencing after 6 months of incubation. These known iron and sulfate reducing populations were also detected in both iron and sulfate reducing media hence confirmed their involvement in reduction of iron and sulfate during 5 months of incubated setups.

## Discussion

Geomicrobiology of AMD including the nature of microorganisms and biogeochemical functions of various acidophilic microorganisms is well established. In contrast to that, the broader ecological roles of AMD organisms in terms of the attenuation of the hazardous nature of such acidic environment remain less explored. Our study demonstrated that it is possible to enhance the activities of indigenous sulfate- and iron-reducing bacteria of an AIS to achieve improvement of its major physicochemical parameters desirable for bioremediation. With respect to the major questions we posed during this study, our results proved that (a) it is possible to enhance the abundance and activities of autochthonous sulfate- and iron-reducing bacteria of an AIS and (b) this altered microbial community could lead toward changing the physicochemical conditions favorably, thus decreasing the hazardous nature of the studied sample considerably.

There are reports highlighting the presence of heterotrophic sulfate- and iron-reducing bacteria (*Clostridiaceae*, *Peptococcaceae*, and *Bacillaceae* members) within highly acidic AMD systems (Sánchez-Andrea et al., 2012a,b). Our biostimulation based approach was successful in enhancing the abundance of *Firmicutes* members capable of anaerobic sulfate-/iron-reduction. In the native AIS, these bacterial taxa constituted only 1.5% which (low abundance of heterotrophic reducing taxa) corroborated the earlier reports on different AMD environments (Chen L.X. et al., 2013; Kuang et al., 2013). Increase in abundance of these anaerobic/facultative anaerobic populations surpassing the acid producing-, sulfur- and metal-oxidizing microorganisms with nutrient amendments was impressive. All these members of the phylum *Firmicutes* were well known for their facultative to strict anaerobic metabolism_,_ but not so much for sulfate- and iron-reduction except few taxa such as *Clostridium*, *Desulfosporosinus*, *Desulfotomaculum* etc. (Chockalingam and Subramanian, 2006; Church et al., 2007; Sánchez-Andrea et al., 2012b; Pan et al., 2017). The increased abundance of gene encoding dissimilatory sulfite reductase (*dsr*B) and *Firmicutes* specific 16S rRNA gene detected in qPCR, reduction of -sulfate/-iron and rise in pH were all in strong agreement. Our results demonstrated that a number of sulfate- and iron-reducing bacterial taxa present in AMD impacted environment can be proliferated and implicated with the desirable reductive processes successfully. The PICRUSt analysis confirmed that the enriched bacterial populations were genetically equipped for dissimilatory sulfate reduction processes.

The effect of cysteine (alone or along with lactate) as successful proxy to provide required metabolic resources and thus biostimulate the target groups of microorganisms could be attributed to its dual characteristics. Cysteine could be used by the microbes as a carbon and nitrogen source, and also might act as a reducing agent (that helps in scavenging the dissolved oxygen) to facilitate reduction of iron and sulfate. Microbes catabolize cysteine for their fermentative mode of metabolism through two enzymes (i) cysteine desulfhydrase which produces NH_3_, pyruvate and H_2_S and (ii) cystathionine-γ-lyase which utilizes an oxidized form of cysteine (Morra and Dick, 1991). Microbe mediated H_2_S production was possible from selective enrichment of soil amended with cysteine through cysteine desulfhydrase enzyme. Wang et al. (2000) demonstrated that the genes encoding cysteine desulfhydrase and serine acetyltransferase may be used to develop a metabolically engineered *Escherichia coli* that can carry out aerobic sulfate reduction. Suitability of carbon sources rich in amino acids, but low in lignin in promoting sulfate reduction was also reported (Coetser et al., 2006). Recently, Zhang et al. (2017) established the role of tryptone and yeast extract in the remediation of mine tailings by promoting the growth of SRB.

Microbial taxa enriched during this study were reported to be of facultative- or strict-anaerobic nature, involved in anaerobic hydrolytic fermentation, cysteine utilization, acetate- and H_2_S-production and metal reduction (Petrie et al., 2003; Church et al., 2007; Finke and Jørgensen, 2008; Kosaka et al., 2008; Li et al., 2011; Bertel et al., 2012; AlAbbas et al., 2013; Hausmann et al., 2016; Peng et al., 2016; Pan et al., 2017). The major genera identified in this study such as *Clostridium*, *Clostridium sensu stricto* members, *Lutispora*, *Sporobacter*, *Acetanaerobacterium*, *Caldicoprobacter*, *Gracilibacter*, *Oxobacter, Fonticella, Papillibacter*, as well as unclassified members of *Ruminococcaceae*, *Lachnospiraceae*, and *Christensenellaceae* were all reported as anaerobic, fermentative, cellulose- and cysteine-metabolizing, acetogenic, and iron reducing members (Grech-Mora et al., 1996; Defnoun et al., 2000; Chen and Dong, 2004; Lee et al., 2006; Shiratori et al., 2008; Bouanane-Darenfed et al., 2011; Chen M. et al., 2013; Fraj et al., 2013; Peng et al., 2016). Presence of these organisms was reported from diverse sulfur-rich environments including hot spring (Zavarzina et al., 2007; Bouanane-Darenfed et al., 2011; Fraj et al., 2013), mine tailings/drainage/soil (Sánchez-Andrea et al., 2011; Gupta et al., 2017), constructed wetland (Lee et al., 2006) and AMD treatments sites (Clarke et al., 2004; Kaksonen et al., 2004; Pruden et al., 2007; Bijmans et al., 2009; Hiibel et al., 2011; Lu et al., 2011; Martins et al., 2011; Sánchez-Andrea et al., 2014; Deng et al., 2016). Considering the known metabolic characteristics of these taxa and their ecological relevance, we could attribute their abundance to the observed sulfate- and iron-reduction. In accordance with previous reports, the other known strict anaerobic sulfate reducing taxa such as *Desulfurispora*, *Desulfotomaculum*, *Desulfosporosinus*, and *Desulfitobacterium* was also enriched during our study (Kaksonen et al., 2004; Church et al., 2007; Hiibel et al., 2008, 2011; Bijmans et al., 2010). The enhanced abundance of facultative anaerobic fermentative and strictly anaerobic sulfate reducing populations following cysteine amendments highlights the synergistic role of these metabolically dependent organisms confirming the fermentation coupled with sulfate reduction phenomenon (Finke and Jørgensen, 2008). We hypothesize that in the presence of cysteine, fermentative organisms become activated, producing metabolites and deplete the dissolved oxygen rapidly and thereby creating more anoxic niches. Within these anoxic micro-niches strict anaerobic populations proliferate, making use of the sulfate as preferred terminal electron acceptor thus facilitates sulfate reduction and rise in pH (Church et al., 2007). Our attempt to confirm the physiological abilities of the enriched populations toward sulfate- and iron-reduction by using culture media specific for *Clostridium* and facultative anaerobic bacteria supported the above hypothesis. We were successful in identifying the facultative and strict anaerobic sulfate- and iron-reducing populations with conformity through specific enrichment and deep sequencing.

The potential involvement of individual members of the enriched populations toward reductive processes was validated by a third level of enrichment wherein sulfate- and iron-reducing populations were grown more selectively in two specific media. These sulfate- and iron-reducing bacteria specific enrichments were meant to segregate and identify the organisms responsible for individual terminal electron acceptor utilization (iron as Fe^3+^ and sulfate as SO_4_^2^). 16S rRNA gene sequencing of metagenomes retrieved from these enrichments revealed that members of the families *Clostridiaceae*, as well as *Bacillaceae* (genera *Lysinibacillus, Bacillus and Paenibacillus* etc.), *Veillonellaceae* and *Ruminococcaceae* etc. specifically contributed toward sulfate- or iron-reduction. Presence of these members in both C_5M and C+L_5M microcosms through DGGE further confirmed their potential of sulfate and iron reduction. *Clostridiaceae* and *Bacillaceae* members were previously reported in different AMD bioremediation studies or in sulfate-/iron-reducing enrichments/AMD environment (Clarke et al., 2004; Scala et al., 2006; Hiibel et al., 2008, 2011; Sánchez-Andrea et al., 2011; Yi et al., 2012; Giloteaux et al., 2013; Zhang and Wang, 2016; Zhang et al., 2016). The predominance of metal reducing *Pelosinus* (member of *Veillonellaceae*) on lactate amendment was reported by Mosher et al. (2012). Metal reduction and fermentative mode of metabolism of *Veillonellaceae* members were reported by earlier investigators including the whole genome sequence analysis of uncultured *Veillonellaceae* strain RU4 that confirmed presence of genes for sulfate reduction as well as polysulfide reduction (Brown et al., 2012; Shah, 2013; Kwon et al., 2016). Zhao et al. (2010) reported the role of *Ruminococcus* spp. (member of *Ruminococcaceae*) in sulfate reduction. Thus in our study, these enriched members confirmed their involvement in iron and sulfate reduction.

## Conclusion

An acidic, sulfate-, iron- and other heavy metal-rich AMD impacted soil harbored low proportion of heterotrophic, sulfate- and iron-reducing anaerobic bacterial populations. These redox active members can be successfully stimulated by cysteine and lactate amendment. These enriched microbial groups can facilitate dramatic change in physiochemical condition. The microorganisms which got enriched with nutrient amendment belonged to the fermentative and strict anaerobic sulfate- and iron-reducing populations affiliated to *Clostridiaceae*, *Veillonellaceae, Bacillaceae*, *Ruminococcaceae* etc. Increased abundance of these organisms as evident from 16S rRNA amplicon sequencing and taxon-specific qPCR; enhancement of *dsr*B gene, change in genomic composition suitable for carrying out the required catabolic function corroborated with reduction in soluble sulfate- and iron-reduction and pH management. This study enabled us to gain a better insight on ecological perspective of the members of phylum *Firmicutes* indigenous to AMD impacted sites and more importantly, their involvement in sulfate- and, iron-reduction processes. The study also demonstrated the suitability of amino acid/protein rich natural substances as potent biostimulation agent for bioremediation of AMD/AMD impacted sites and provided us the specific microbial populations capable of anaerobic sulfate-, and/or iron-reduction which could be used as a potent bioaugmentation agent for future bioremediation applications.

## Author Contributions

PS conceived and designed the experiments and arranged funds. AG performed the major experiments. PS and AG were responsible for manuscript preparation. MP, PS, and AG arranged sampling from MCP. AG and JS performed the qPCR. AG and AD performed the bioinformatics analysis for deciphering microbial diversity. AG and AD performed the 16S rRNA gene amplicon sequencing in Ion S5 sequencer.

## Conflict of Interest Statement

The authors declare that the research was conducted in the absence of any commercial or financial relationships that could be construed as a potential conflict of interest.

## Footnotes

1 www.ezbiocloud.net

2 www.arb-silva.de/documentation/release-119

3 www.arb-silva.de/documentation/release-119

4 http://www.interactivenn.net

5 https://huttenhower.sph.harvard.edu/galaxy

6 http://greengenes.secondgenome.com
