# Supplementary figures and images for: Low-Abundance Members of the Firmicutes Facilitate Bioremediation of Soil Impacted by Highly Acidic Mine Drainage From the Malanjkhand Copper Project, India
Source: Front Microbiol. 2018 Dec 11;9:2882. doi: 10.3389/fmicb.2018.02882 (PMC6297179; doi:10.3389/fmicb.2018.02882)

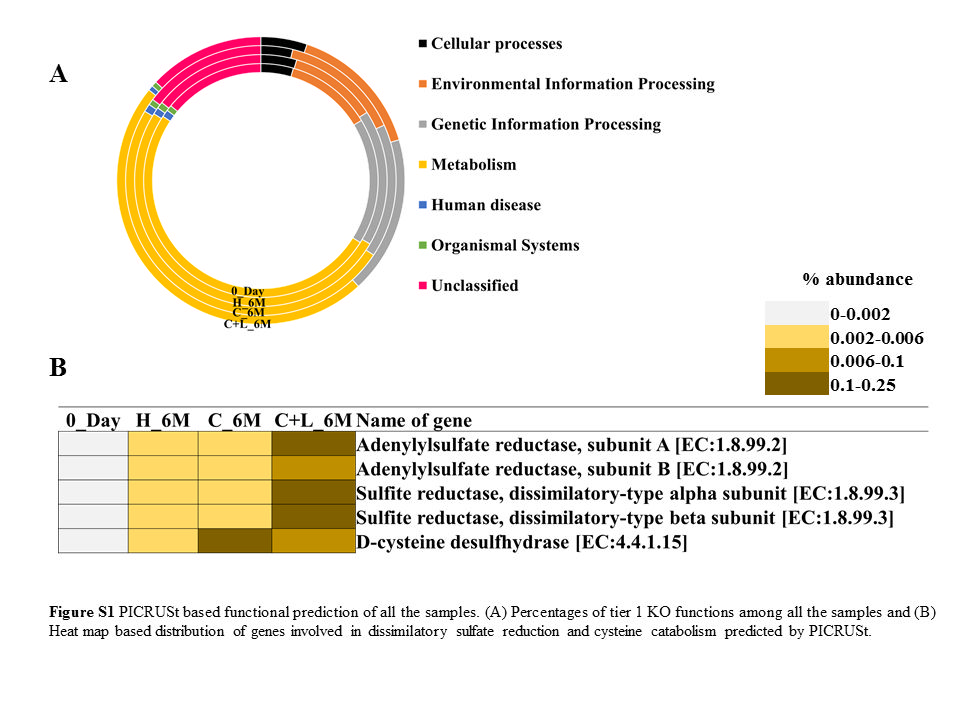

Supplement: Supplementary file 1 [file Image_1.TIF]

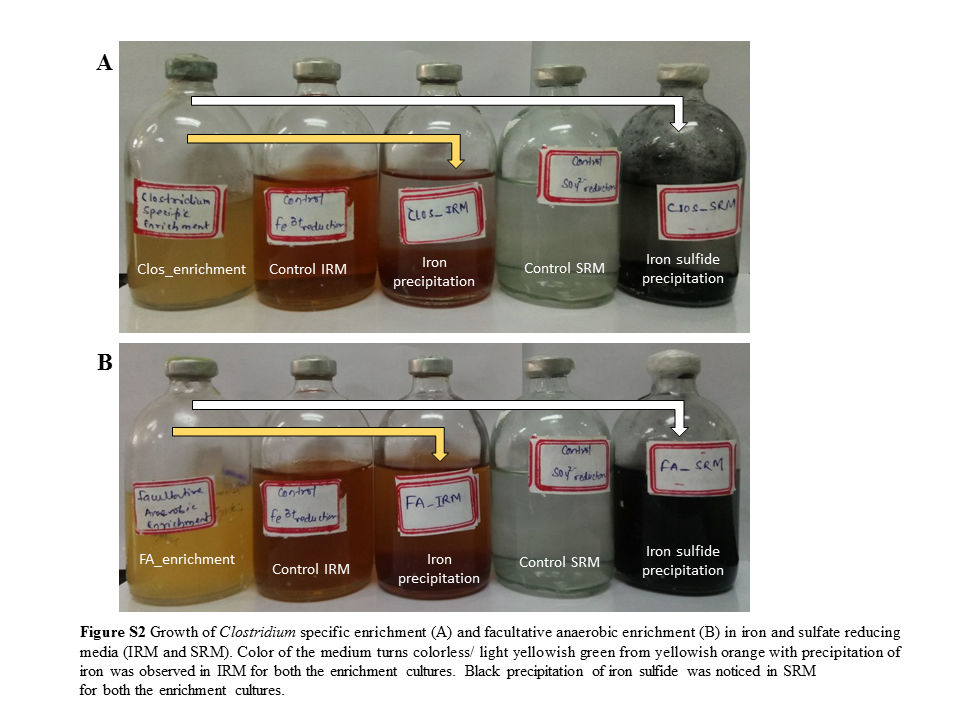

Supplement: Supplementary file 2 [file Image_2.TIF]
